# Comparative genome-wide methylation analysis reveals epigenetic regulation of muscle development in grass carp (*Ctenopharyngodon idellus*) fed with whole faba bean

**DOI:** 10.7717/peerj.14403

**Published:** 2022-11-21

**Authors:** Yichao Li, Bing Fu, Junming Zhang, Guangjun Wang, Jingjing Tian, Hongyan Li, Yun Xia, Jun Xie, Ermeng Yu

**Affiliations:** Pearl River Fisheries Research Institute of CAFS, Guangzhou, China

**Keywords:** Faba bean, DNA methylation, Crisp grass carp, Muscle development

## Abstract

Crisp grass carp (CGC), the most representative improved varieties of grass carp (GC), features higher muscle hardness after feeding faba bean (*Vicia faba* L.) for 90–120 days. DNA methylation, a most widely studied epigenetic modification, plays an essential role in muscle development. Previous studies have identified numerous differentially expressed genes (DEGs) between CGC and GC. However, it remains unknown if the expression levels of these DEGs are influenced by DNA methylation. In the present study, we performed a comprehensive analysis of DNA methylation profiles between CGC and GC, and identified important candidate genes related to muscle development coupled with the transcriptome sequencing data. A total of 9,318 differentially methylated genes (DMGs) corresponding to 155,760 differentially methylated regions (DMRs) were identified between the two groups under the CG context in promoter regions. Combined with the transcriptome sequencing data, 14 key genes related to muscle development were identified, eight of which (gsk3b, wnt8a, wnt11, axin2, stat1, stat2, jak2, hsp90) were involved in muscle fiber hyperplasia, six of which (tgf-β1, col1a1, col1a2, col1a3, col4a1, col18a1) were associated with collagen synthesis in crisp grass carp. The difference of methylation levels in the key genes might lead to the expression difference, further resulting in the increase of muscle hardness in crisp grass carp. Overall, this study can help further understand how faba bean modulates muscle development by the epigenetic modifications, providing novel insights into the texture quality improvement in other aquaculture fish species by nutritional programming.

## Introduction

DNA methylation, the most widely studied epigenetic regulatory mechanisms, is important for the epigenetic regulation of gene expression and involved in the regulation of muscle development (Jones, 2012). A comparative analysis of whole genome DNA methylation regulation of gene expression the level of transcription in muscles of Japanese Black and Chinese Red Steppes cattle identified several genes associated with differentially methylated regions (DMRs) that is related to muscle development (Fang et al., 2017). Whole Genome DNA methylation patterns in the skeletal muscle of sheep showed that some key differentially methylated genes (DMGs) were involved in regulating skeletal muscle development and fatty acid metabolism (Fan et al., 2020). A comprehensive epigenome atlas in muscles of Landrace pigs revealed DNA methylation affected the expression of muscle-related genes by modulating the accessibly of upstream myogenesis TF binding, further regulating skeletal myogenesis (Yang et al., 2021). These studies indicated that DNA methylation has important regulatory roles in muscle development.

Also noteworthy, there are growing evidences that epigenetic modifications, DNA methylation in particular, can be influenced by external environmental stimuli (including dietary factors), thereby affecting gene expression and causing phenotypic changes (Guo, Luo & Lin, 2020). However, previous studies mainly focus on how dietary factors contribute to changes in metabolic phenotypes via DNA methylation variation (Park et al., 2017). For instance, the changing carbohydrate content of trout diet can influence DNA methylation level of gluconeogenic gene, promoting the initialisation of the glucose intolerant phenotype (Marandel et al., 2016). High-carbohydrate diet can change DNA methylation pattern in metabolic pathways, contributing to hyperglycemia and fat deposition in grass carp (Cai et al., 2020). Up to now, no studies have established a relationship between nutritional status and the epigenetic regulation of muscle development, through the changes of DNA methylation status.

Faba beans (*Vicia faba L*.), one of the most important legume, is traditionally used as a rich source of plant protein, nutrients and dietary fiber for human nutrition in the African, Indian, Chinese, English, Mediterranean and Middle Eastern areas (Rahate, Madhumita & Prabhakar, 2021). The presence of various bioactive compounds in faba bean has been proven protective effects for several chronic diseases like cancer, diabetes type 2, cardiovascular diseases, Parkinson’s disease, *etc*. (Rahate, Madhumita & Prabhakar, 2021; Rabey et al., 1992). Surprisingly, previous studies have found that faba bean improves the muscle texture through the changes in muscle structure (Yu et al., 2014; Cutrignelli et al., 2008). This phenomenon was firstly observed in grass carp. In the early 1970s, faba bean has been discovered to improve the muscle textural of grass carp as an aquafeed in Guangdong Province, China, and this value-added carp was termed as crisp grass carp (Ma et al., 2020). Unlike ordinary grass carp, crisp grass carp features significant increased muscle textural quality (hardness, springiness, *etc*.), and its fillet products are exported globally to foreign countries and regions such as North and Latin America as well as Southeast Asia (Yu et al., 2017). Previous studies have shown that the muscle hardness of crisp grass carp is well associated with the expression of muscle fiber and collagen synthesis genes (Yu et al., 2014). However, epigenetic regulatory mechanisms for these genes in crisp grass carp remain unclear. Therefore, exploration of DNA methylation profiles in muscle of crisp grass carp would help understand how dietary factors (faba bean) modulates muscle development by the epigenetic modifications.

In this study, we used whole-genome bisulfite sequencing (WGBS) to carry out genome-wide DNA methylation analysis of crisp grass carp and ordinary grass carp and profiled their methylation patterns. Then, we performed an association analysis between differentially methylated genes (DMGs) and differentially expressed genes (DEGs) obtained from our previous transcriptome data to identify 34 key genes involved in the regulation of muscle texture in crisp grass carp. This research provides novel insights into the texture quality improvement in other aquaculture fish species by nutritional programming.

## Materials and Methods

### Feeding trial

Healthy grass carp were purchased from an aquaculture farm in Zhongshan, Guangdong Province, China. The fish were first temporarily cultured in a cement pond (5 m × 5 m × 1.5 m) for 1 week and the feed amount for each day was 2–3% of fish weight. A total of 90 fish with initial weight of 742 ± 55 g (mean ± SD) were randomly divided into crisp grass carp and ordinary grass carp groups, with three replicates each group. They were cultured in six cement ponds (2 × 2 × 1.5 m), with 15 fish in each pond. Crisp grass carp were fed solely with whole broad beans and ordinary grass carp were fed with commercial diet. The feed amount for each day was 3–5% of fish weight and the fish were fed at 8:00 am and 5:00 pm each day for 120 days. The water temperature was kept at 25–30 °C, pH was 6.5–7.5, and dissolved oxygen was above 5.0 mg/L. After 120 days, the final weights of crisp grass carp and ordinary grass carp were 1,582 ± 75 g and 1,957 ± 212 g, respectively.

### Sampling procedures

Three individuals randomly captured from each group were placed into three big plastic containers (1.5 m × 1.5 m × 1 m) and euthanized with pH-buffered tricaine methanesulfonate (250 mg/L) following the procedures approved by the Malmö-Lund Ethical Committee. Once fin and operculum movement ceased, muscle tissues (2 g) were washed repeatedly with sterile saline and snap-frozen in liquid nitrogen and stored at −80 °C for RNA and whole-genome bisulfite sequencing. Some muscle samples were collected for the measurement of textural quality parameters (hardness, chewiness, gumminess, springiness), determination of collagen content, and haematoxylin-eosin (H&E) staining.

The experimental protocols used in this study were approved by the Laboratory Animal Ethics Committee of Pearl River Fisheries Research Institute, CAFS, China, under permit number LAEC-PRFRI-20210149.

### Measurement of textural quality parameters and H&E staining of skeletal muscle

The textural parameters were measured using a Universal TA Texture Analyzer (Tengba, China) with fillets taken from the back muscle (Ma et al., 2020). For the determination of muscle fiber density, the number of muscle fibers within the field were calculated using the DP2-BSW 2.2 software (Olympus, Tokyo, Japan). Muscle fiber diameter was calculated by assuming that muscle fibers are cylindrical, and thus the diameter was calculated according to s = πr^2^ (where s is the muscle fiber area and r is the muscle fiber radius) (Lee & Mcpherron, 2001). Collagen content of muscle was determined by Kit No. YX-E21992F (the Ultra-Sensitive Fish ELISA Kits). Muscle blocks (3 mm × 3 mm × 3 mm) fixed in 10% formalin were used for haematoxylin-eosin (H&E) staining according to the standard histology protocol.

### RNA extraction, transcriptome library preparation, and illumina sequencing

The method to obtain RNA-seq data have been clearly described in previous study (Tian et al., 2020). Briefly, the muscles from three fish per group were employed to extract total RNA using RNAiso Plus (TaKaRa, Dalian, P.R. China) according to the manufacturer’s instructions. After determining the concentration and purity of RNA by the ratio of A260 to A280 (A260:280 ≥ 1.8 and ≤ 2.0), RNA integrity was evaluated using an Agilent 2100 Bioanalyzer Lab-on-chip system (Agilent Technologies, Santa Clara, CA, USA). Then, the total RNA samples were treated with DNase I to degrade any possible DNA contamination, followed by the mRNA enrichment using the magnetic beads with Oligo (dT). Next, the mRNA was fragmented into short pieces by mixing with fragmentation buffer. Subsequently, after synthesizing the first strand of cDNA using random hexamer-primer, Buffer, DNA polymerase I, dNTPs, and RNase H were used to synthesize the second strand. The cDNA fragments were purified using magnetic beads, and then End reparation and 3′-end single nucleotide A (adenine) was addicted. Lastly, sequencing adaptors were ligated to fragments, which were enriched using PCR amplification. For QC step, ABI StepOnePlus Real-Time PCR System and Agilent 2100 Bioanalyzer were employed for quantification and qualification of the sample library. The library products were sequenced using Illumina HiSeqTM 2000.

### Transcriptome assembly, identification of differentially expressed genes (DEGs)

Raw reads were cleaned by removing unqualified reads, including those containing adapters or over 50% low-quality (Q-value ≤ 5) bases, with over 10% unknown nucleotides ‘N’. Then, the reads were mapped to grass carp genome (Li & Durbin, 2009). Subsequently, the correlation value between each sample pair was calculated according to the FPKM results. Finally, the DEGs were determined according to expression profiles and following criteria: (1) the change in gene expression levels in CDFG versus FBFG was | log2 ratio| > 1, (2) False Discovery Rate (FDR) ≤ 0.001. The RNA-sqe data have been deposited in the Sequence Read Archive (SRA) under accession number: PRJNA548646.

### DNA extraction and whole-genome bisulfite sequencing

Genomic DNA was isolated according to the method in File S1. Purified DNA were sent to BGI (BGI Tech Co., Ltd., Shenzhen, China) for library constructing and sequencing. Firstly, the genomic DNA was fragmented to a mean size of approximately 250 bp with a Bioruptor (Diagenode, Liege, Belgium), followed by end repair, dA addition to 3′-end, and adaptor ligation. Subsequently, ligated DNA was bisulfite converted using the ZYMO EZ DNA Methylation-Gold kit. Fragments were excised from the same lane of a 2% TAE agarose gel. Products purified with QIAquick Gel Extraction kit (Qiagen) were amplified by PCR. Lastly, qualified fragments were sequenced using the HighSeq4000. The sequenced raw data was deposited at the NCBI Short Reads Archive (SRA) under accession number PRJNA835315.

### Data filtering and reads alignment

Following sequencing, the data of raw reads were filtered to guarantee the quality by FASTX-Toolkit (http://hannonlab.cshl.edu/fastx_toolkit/), including removing low-quality reads which meet any one of the two conditions: (1) Unknown bases are more than 10%; (2) The ratio of bases whose quality was less than 20 was over 10%. After filtering, the bisulfite-treated reads were mapped to the reference genome of grass carp (Wang et al., 2015) by BSMAP (Bisulfite Sequence Mapping Program). And then remove the duplication reads and merge the mapping results according to each library. Reads Alignment process is as follows: BSMAP parameters for PE reads: BSMAP -a filename_1.clean.fq.gz-bfilename_2.clean.fq.gz-ofilename.sam-dref.fa.-u-v8-z33-p4-n0-w20-s16-f10-L100. If use Hiseq2000, -z should be set 64; samtools parameters: samtools view -S-b -o filename.bam filename.sam samtools sort -m 2000000000 filename.bam filename.sort samtools index filename.sort.bam. Then, sequence reads were transformed to fully bisulfite-converted versions (C-to-T and G-to-A) and aligned with converted versions of the genome. The mapping rate and bisulfite conversion rate of each sample were calculated as follows: Bisulfite Conversion Rate = 1 − methylation rate of Lambda DNA. More detailed methods and parameters have been described in File S3.

### Differentially methylated regions analysis

DNA methylation level was calculated by dividing the number of reads covering each mC by the total reads covering that cytosine (Lister et al., 2009), which was also equal the mC/C ratio at each reference cytosine (Xiang et al., 2010). DMRs were detected by the following formula: Rmaverge = Nmall/Nmall + Nnmall (Nm meant the reads of mC, while Nnm represents non-methylation reads). Putative DMRs were identified by comparison between two group of grass carp methylomes using windows which contained at least five CpG sites with a two-fold change in methylation level and *P* value ≤ 0.05 (Fisher test).

### GO and KEGG enrichment analysis of DMR-related genes

Gene Ontology (GO) and Kyoto Encyclopedia of Genes and Genomes (KEGG) pathway analyses were carried out in the promoter regions. DMRs were mapped to GO terms in the database (http://www.geneontology.org/), and the gene numbers for every term were calculated. Then, significantly enriched GO terms were identified on GO TermFinder (https://www.yeastgenome.org/goTermFinder). KEGG pathways analysis were performed with KOBAS software (version 2.0) (Mao et al., 2005). GO terms and KEGG with corrected *p* ≤ 0.05 were considered to be significantly enriched.

### Protein-protein interaction network analysis of differentially methylated and expressed genes related to muscle structure and development

To further explore the molecular mechanisms underlying the muscle textural improvement in crisp grass carp, 34 overlap genes between DMGs and DEGs associated with muscle textural improvement were obtained with following criteria: (1) genes were differentially methylated and expressed between two groups; (2) genes were enriched in pathways associated with muscle structure and development; (3) genes were differentially expressed in crisp fish fed with faba bean reported by previous studies (Yu et al., 2014, 2017, 2019, 2020; Xu et al., 2020; Tian et al., 2020; Fu et al., 2022; Song et al., 2022); (4) genes were reported to be associated with muscle structure and development of fish. The application of these criteria resulted in the identification of 34 candidate genes related to muscle structure and development of crisp grass carp. Differentially methylated and expressed genes were selected for the construction of protein-protein interaction network (PPI). Specifically, the genes lists were imported into STRING Version 11.0 (https://string-db.org/), a bioinformatics analysis tool for obtaining protein connection scores. Then, the obtained combination scores were rescaled into the confidence range (0.0–1.0) to connect all the scores. STRING filters out protein interactions according to following conditions: (1) Default String confidence scores <0.400 represent low confidence, (2) 0.400–0.700 represent medium confidence, (3) >0.700 represents high confidence. To build stringent and comprehensive interactions, medium confidence protein-protein interactions (String confidence scores ≥0.400) was employed as a cut-off filter. Lastly, the interaction scores and relationships of proteins in two groups were extracted as a text-delimited file based on the zebrafish database. The PPI network was visualized using Cytoscape v. 3.8.2 software (Klein, Sun & Staff, 2019).

### Data analysis

The comparisons were analyzed by using the Student *t*-test with SPSS software (version 19.0). GraphPad Prism 9 software was applied to generate the box figures. The *P*-value <0.05 was considered as statistically significant, and the *P* and F values were accurately calculated (95% confidence levels). The results are expressed as “mean ± SE”. Statistical results are fully reported in File S2, including degrees of freedom, the exact F values, effect size and *P*-value.

## Results

### Textural quality of skeletal muscle

**Figure 1:**
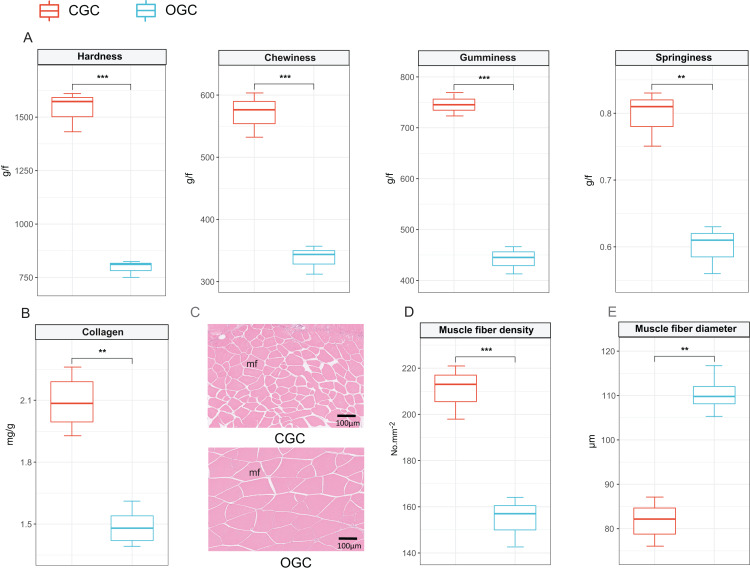
Textural quality of ordinary grass carp (OGC) and crisp grass carp (CGC) muscle. (A) Muscle textural parameters. (B) Collagen content. (C) Microstructural observation(×400). mf, muscle fiber. (D) Muscle fiber density. (E) Muscle fiber diameter. Statistical analyses were performed using Student’s *t*-test, n = 3, **P < 0.01, ***P < 0.001.

The contents of hardness, chewiness, gumminess, and springiness in crisp grass carp muscle were higher than those in ordinary grass carp (*P* < 0.01) (Fig. 1A). Collagen content of crisp grass carp muscle was higher than that of ordinary grass carp (Fig. 1B). The transverse microstructure diagrams of grass carp are shown in Fig. 1C. Statistical analysis showed that muscle fiber densities of crisp grass carp (146.0 ± 1.0 No./mm^2^) were higher than that of the ordinary grass carp (83.0 ± 3.0 No./mm^2^), and muscle fiber diameters of FB group (87.33 ± 0.87 μm) were lower than that of the control group (107.00 ± 1.17 μm) (*P* < 0.05) (Figs. 1D and 1E). The raw data of textural quality is provided as File S2.

### Global mapping and statistical analysis of the WGBS reads

To obtain the DNA methylation levels of muscle in crisp grass carp and ordinary grass carp, we conducted whole-genome bisulfite sequencing (WGBS). After quality control of filtering, approximately 2.07 billion clean reads were obtained, composed of 350.17, 320.22, and 334.57 million reads for each crisp grass carp sample and 351.59, 318.22, and 393.14 million reads for each ordinary grass carp sample (Table 1). We read approximately 75.53 G raw bases and 81.6 G clean bases in total. More than 95% of the raw reads were clean reads (Table 1). Of these clean reads, more than approximately 93% were mapped to the reference genome of grass carp. The bisulfite conversion rate (%) of all sequencing libraries were about 96%.

**Table 1:** Alignment statistics with reference genome.

Sample ID	Clean reads	Clean bases (G)	Clean ratio (%)	Mapped reads	Mapping rate (%)	Uniquely mapped reads	Uniquely mapping rate (%)	Bisulfite conversion rate (%)
CGC1	350,174,586	26.50	95.97	325,098,027	92.84	309,905,822	88.5	99.05
CGC2	320,220,488	24.01	97.46	298,520,528	93.22	284,589,492	88.87	99.05
CGC3	334,573,094	25.02	96.13	311,321,665	93.05	296,207,252	88.53	99.02
OGC1	351,592,970	26.01	96.50	327,220,334	93.07	312,048,923	88.75	99.06
OGC2	318,216,916	23.70	96.37	296,111,680	93.05	282,023,990	88.63	99.05
OGC3	393,144,866	28.30	96.78	365,808,412	93.05	348,424,438	88.62	99.05

**Note:**

CGC, Crisp grass carp; OGC, Ordinary grass carp, DGC, domesticated grass carp; WGC, wild grass carp.

### Global DNA methylation patterns of crisp grass carp and ordinary grass carp

**Figure 2:**
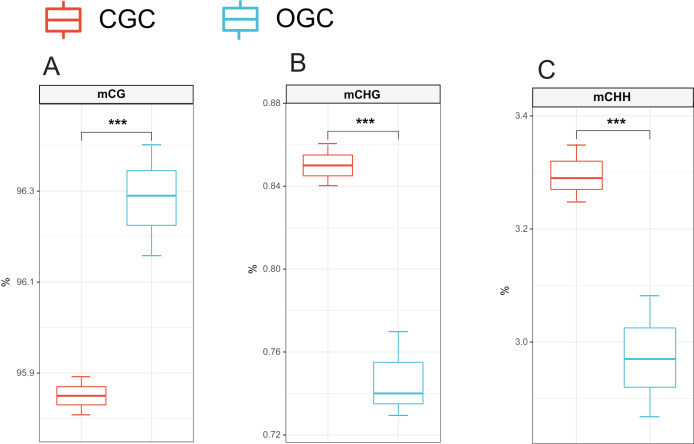
Proportions of CG, CHG and CHH in all Methyl-cytosine. (A) mCG. (B) mCHG. (C) mCHH. CGC, crisp grass carp; OGC, ordinary grass carp. CG, CHG, and CHH (where H is A, C, or T). Statistical analyses were performed using Student’s *t*-test, n = 3, ***P < 0.001.

The methylation levels of the whole genome were listed in Fig. 2. Among the detected methylation sites, mCG sites accounted for approximately 96%; mCHG and mCHH only accounted for approximately 0.8% and 3%, respectively. The methylation level of CG was significantly higher than that of CHG and CHH (*P* < 0.05). Compared to ordinary grass carp, the proportion of mCG was significantly lower (*P* < 0.05), whereas proportion of mCHG and mCHH in the crisp grass carp was significantly higher than that in the ordinary grass carp (*P* < 0.05) (Table 2).

**Table 2:** Proportions of CG, CHG and CHH in all Methyl-cytosine.

		mCG	mCHG	mCHH
CGC1	mC number	26,421,894	237,327	906,008
	Proportions (%)	95.85	0.86	3.29
CGC2	mC number	26,274,536	236,120	889,033
	Proportions (%)	95.89	0.86	3.25
CGC3	mC number	26,435,931	231,192	924,601
	Proportions (%)	95.81	0.84	3.35
OGC1	mC number	26,275,999	202,362	810,165
	Proportions (%)	96.29	0.74	2.97
OGC2	mC number	26,116,512	208,134	835,541
	Proportions (%)	96.16	0.77	3.08
OGC3	mC number	26,564,745	201,726	790,038
	Proportions (%)	96.40	0.73	2.87

**Note**:

CGC, crisp grass carp; OGC, ordinary grass carp. CG, CHG, and CHH (where H is A, C, or T); DGC, domesticated grass carp; WGC, wild grass carp.

**Figure 3:**
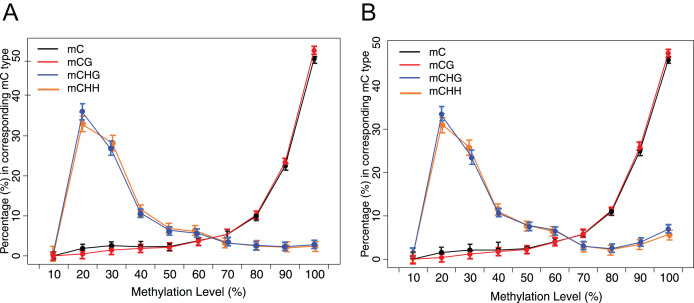
Distribution of methylation level of mC in each sequence context. (A) Crisp grass carp (CGC). (B) Ordinary grass carp (OGC). The x-axis was defined as the percentage of reads mC at a reference cytosine site. The y-axis indicated the fraction of total mC calculated within bins of 10%.

Methylation status of CG, CHG and CHH of grass carp genome showed the methylome’s overall characteristics (Fig. 3). The methylation levels of about 25% of mCG were hypermethylated (methylation level > 90%). However, only approximately 2% of mCHH and mCHG were hypermethylated. The methylated Cs mainly occur under CG context, followed by CHH and CHG.

### DNA methylation levels of different genomic features

**Figure 4:**
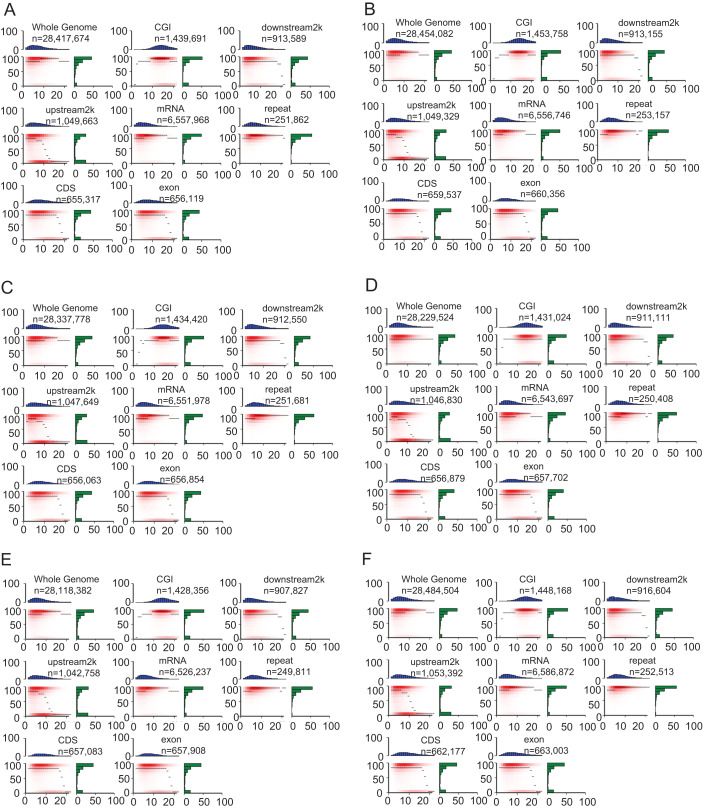
Heat maps show distinct methylation and CpG density patterns. Crisp grass carp (CGC), Ordinary grass carp (OGC). (A) Sample CGC1. (B) Sample CGC2. (C) Sample CGC3. (D) Sample GC1. (E) Sample GC2. (F) Sample GC3. Heat maps of distinct methylation and CpG density patterns. CpG density (x-axis) was defined as numbers of CpG dinucleotides in 200 bp windows. Methylation level (y-axis) was defined as average methylation level of cytosines in CpGs. The thin black lines within each heat map denoted the median methylation level of CpGs at the given local density. The red color gradient indicated abundance of CpGs that falled into bins of given methylation levels and CpG densities. The blue bar charts above each heat map showed the distribution of CpG densities, projected onto the x-axis of the heat maps. The green bar charts to the right of the heat maps show the distribution of methylation levels, projected onto the y-axis of the heat maps.

The heatmap demonstrated the methylation landscape in different genomic features, including whole genome, CGI, downstream 2 kb, upstream 2 kb, mRNA, repeat, CDS, exon, and provide additional information of these components (Fig. 4). Compared with other genomic features, CpG islands contained the highest numbers of CpG sites (about 10–20 CpG sites in a 200 bp window). Approximately 60% of CpG sites in CpG islands were hypermethylated (methylation levels >90%) in the heatmap (Fig. 4). However, the other genomic features generally contained 0–10 CpG sites in the 200 bp window, A smaller portion of CpG sites within whole genome, mRNA and repeat were hypomethylated (methylation levels <10%) compared with that of remaining genomic features (CGI, downstream 2 kb, upstream 2 kb, CDS, exon).

### DNA methylation patterns across the entire transcriptional units at whole genome level

**Figure 5:**
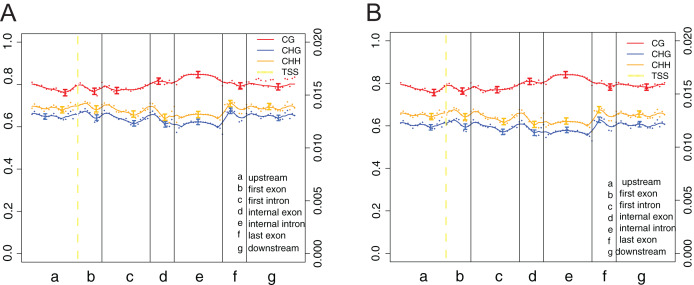
DNA methylation patterns across the entire transcriptional units at whole genome level. (A) Crisp grass carp (CGC). (B) Ordinary grass carp (OGC). The canonical gene structure was defined by seven different features, denoted by the x-axis. The length of each feature was normalized and divided into equal numbers of bins. Each dot denoted the mean methylation level per bin and the respective lines denoted the 5-bin moving average. Each feature was analyzed separately for the numbers listed in the table below the figure. The green vertical line indicated the mean location of the transcription start sites.

The canonical DNA methylation patterns across the entire transcriptional units (upstream, first exon, first intron, *etc*.) at the whole-genome level can help study the changes of methylation levels in different features, contributing to reveal the relationship between DNA methylation profiles and genes expression (Fig. 5). Obviously, methylation levels under cytosine context (CG) were higher than those of CHG and CHH across the entire transcriptional units (Fig. 5). Besides, a modest elevation in methylation level was found at internal exons and internal intron during transcriptional unit scanning. The lowest methylation level occurs in the first intron, followed by the last exon and downstream.

### Identification and enrichment analysis of differential methylated regions

To further characterize the differences of genome methylation levels between crisp grass carp and ordinary grass carp, differentially methylated regions (DMRs) and differentially methylated genes (DMGs) were identified. The results showed that a total of 155,760 DMRs were identified between the two groups under the CG context, which corresponded to 9,318 DMGs in promoter regions (Table S1).

**Figure 6:**
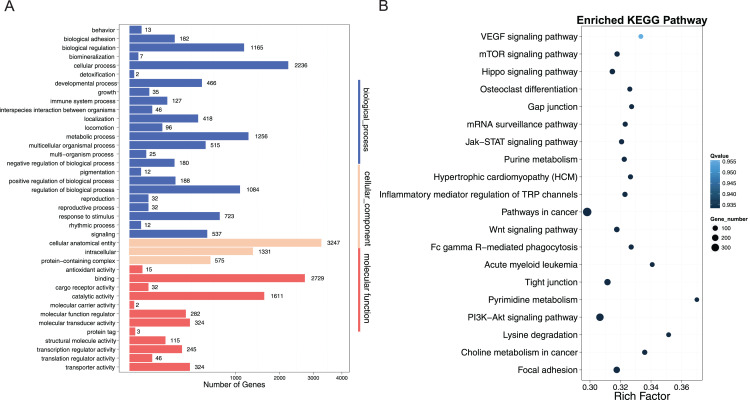
GO and KEGG pathway analysis of DMRs-related genes in promoter regions. (A) GO analysis of differentially methylated regions (DMRs)-related genes in promoter region; The x-axis represented three domains of GO and the y-axis represented the gene number in each pathway and process. (B) Pathway analysis of DMRs-related genes in promoter region; The abscissa represented the richness factor, and the ordinate represented the enriched pathway terms. Q-value represented the corrected *P*, and a small Q-value indicated high significance.

To better understand the role of DNA methylation on muscle development in crisp grass carp, we performed GO and KEGG pathway analyses of DMGs (CG context) to provide significantly enriched GO terms corresponding to specific biological process, cellular component, and molecular function. Our results showed that 39 GO terms were significantly enriched in promoter regions (corrected *P*-value < 0.05), 24 of which were classified as biological process. Specifically, in promoter regions, the DMGs were involved in cellular anatomical entity, binding, cellular process, catalytic activity, intracellular, metabolic process and other process (Fig. 6A). KEGG pathway analysis, which is an alternative approach to categorize gene functions, was also performed for the DMGs in promoter regions. DMGs were significantly enriched in pathways related to muscle growth, including PI3K−Akt signaling pathway, focal adhesion, tight junction, Wnt signaling pathway, Jak−STAT signaling pathway (Fig. 6B).

### Association analysis of differentially methylated genes (DMGs) and the differentially expressed genes (DEGs)

**Figure 7:**
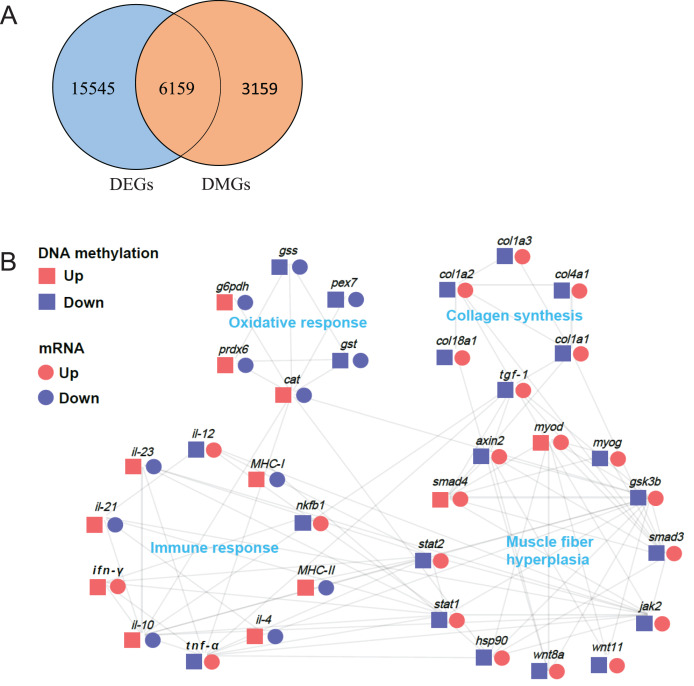
Protein-protein interaction network analysis of differentially methylated and expressed genes related to muscle structure and development. (A) Venn analysis of DMR-related genes (DMGs) and transcriptome differentially expressed genes (DEGs). (B) Protein-protein interaction network analysis of genes related to muscle structure and development. The log_2_ (fold change) values were used to create the histogram.

DNA methylation in promoter regions can lead to decrease in gene expression level. To investigate the relationship between these DMGs and the DEGs obtained from our previous transcriptome data (Tian et al., 2020), we performed an association analysis of the DMGs in promoter regions and DEGs data (Table S2). First, we arranged all DMGs and DEGs for Venn analysis. As a result, we observed 6,159 overlapped genes (Fig. 7A). Furthermore, we identified 34 genes related to muscle structure and development, including *myod* (myogenic differentiation antigen), *myog* (myogenin), *hsp90* (heat shock protein HSP 90), *smad3* (SMAD family member 3), *smad4* (SMAD family member 4), *gsk3b* (glycogen synthase kinase 3 alpha), *wnt8a* (Wnt Family Member 8A), *wnt11* (Wnt Family Member 11), *axin2*, *stat1* (signal transducer and activator of transcription 1), *stat2* (signal transducer and activator of transcription 2), *jak2* (Janus kinase 2), *tgf-β1* (transforming growth factor β1), *col1a1* (collagen type I alpha 1), *col1a2* (collagen type I alpha 2), *col1a3* (collagen type I alpha 3), *col4a1* (collagen type IV alpha 1), *col18a1* (collagen type XVIII alpha 1), *g6pdh* (6-phosphogluconate dehydrogenase), *gst* (glutathione S-transferase), *gss* (glutathione synthase), *cat* (catalase), *prdx6* (peroxiredoxin-6), *pex7* (peroxisomal biogenesis factor 7), *nfkb1* (Nuclear Factor Kappa B Subunit 1), *tnf-α* (tumor necrosis factor-alpha), *ifn-γ* (interferon gamma), *il-4* (interleukin-4), *il-10* (interleukin-10), *il-12* (interleukin-12), *il-21* (interleukin 21), *il-23* (interleukin-23), *MHC-I* (MHC Class I), *MHC-II* (MHC Class II). The protein-protein interaction network analysis demonstrated that these highly correlated genes were composed of four parts: muscle fiber hyperplasia, collagen synthesis, oxidative response and immune response (Fig. 7B).

## Discussion

Faba bean has been reported to improve fish textural quality, such as grass carp and tilapia and Yellow River carp (Yu et al., 2014; Lun et al., 2007; Song et al., 2022). Previous studies demonstrated that faba bean can enhance textural parameters (hardness, chewiness, gumminess and springiness) mainly *via* increasing muscle fiber density and collagen content (Ma et al., 2020). The results in this study are consistent with the previous findings. Muscle growth and development are regulated at the cellular and genomic levels and influenced by epigenetic modification *via* DNA methylation and demethylation (Zanou & Gailly, 2013; Carrio & Suelves, 2015). Previous study has confirmed that numerous genes and pathways related to muscle growth and development were differentially expressed between the CGC and OGC through transcriptome analysis (Yu et al., 2014; Xu et al., 2020). Therefore, the genome-wide DNA methylation profile of sheep muscle were investigated by WGBS to elucidate the relationship between muscle textural improvement and DNA methylation. DNA methylation is considered to be most important epigenetic regulatory mechanisms, and a growing number of studies have revealed that DNA methylation play an important role in regulating muscle development in different species (Yang et al., 2021). Also, there is growing evidence that dietary factors could affect gene expression and cause phenotypic changes *via* DNA methylation variation, but most these genome-wide DNA methylation analysis has been performed for specific types of metabolism in different breeds (Park et al., 2017). No studies have built the links between dietary factors and muscle development through change of DNA methylation. In the present study, we systematically reported a DNA methylation patterns in the muscle of crisp grass carp and ordinary grass carp, and performed an association analysis between differentially methylated genes (DMGs) and differentially expressed genes (DEGs) to identify important genes, further uncovering how dietary factors (faba bean) mediate muscle development in crisp grass carp by DNA methylation variation.

The overall genome-wide methylation patterns were different between two groups. Our results showed that the cytosine methylation levels under CG context were much higher than those under CHG and CHH contexts, which was consistent with evidence reported in grass carp and other fish species (Cai et al., 2020; Pan et al., 2021). Through the analysis of DNA methylation levels across the distinct genomic features and entire transcriptional units, we found that about 80% of CG was methylated in all regions, which is consistent with previous finding in grass carp (Cai et al., 2020). However, the methylation patterns of grass carp were different from those of zebrafish and hybrid tilapias (*O. niloticus × Oreochromis mossambicus*) (~86% and 69.60% of cytosines in CG context, respectively) (Akemann et al., 2018; Wan et al., 2016). Furthermore, the lowest methylation level mainly occurs in the first exons, followed by the last exons and the internal exons, which is also consistent with previous study (Cai et al., 2020), suggesting that mutagenic effects appear to occur at the first exons (Yang et al., 2020). In contrast, internal intron is more likely to be influenced by the regulatory impact of DNA methylation and might play a role in constructing alternative splicing.

By overlapping the differentially methylated regions and functional gene regions, 9,318 DMGs were identified in this study. To reveal the role of DMGs with functional annotations, KEGG pathway enrichment analysis was used to identify the pathways of differentially methylated genes in the muscle of CGC and OGC. In present study, numerous genes were enriched in seven pathways related to muscle structure and development in promoter regions were confirmed, including PI3K-Akt signaling pathway (286 genes), MAPK signal pathway (213 genes), focal adhesion (201 genes), Hippo signaling pathway (118 genes), Wnt signaling pathway (121 genes), mTOR signaling pathway (102 genes), Jak-STAT signaling pathway (93 genes). Watt et al. (2018) reported that The PI3K-Akt signaling pathway plays an important role in muscle growth and development *via* regulating muscle protein synthesis and degradation (Zhang et al., 2016). In present study, the top enriched differentially methylated genes were related to the PI3K-Akt signaling pathway with about 286 genes enriched, suggesting their essential roles in regulating muscle proliferation and differentiation (Elia et al., 2007). As previously reported, MAPK signal pathway plays an essential role in early myogenesis and somite development by activating regulatory factors (*e.g*., MyoD) (Keren, Tamir & Bengal, 2006). In this study, above 200 DMGs were observed in MAPK signal pathway, indicating their critical roles in the regulation of muscle structure and development. Furthermore, focal adhesion has been found to participating in the regulation of specific developmental states in myoblast differentiation and muscle fiber formation *via* inducing change of focal adhesion kinases (Graham, Gallagher & Cardozo, 2015). In this study, the focal adhesion pathway was identified with 201 DMGs between the two types of muscles, indicating its crucial role in regulating muscle proliferation and differentiation. Hippo signaling pathway, a key element of muscle fibers and muscle satellite cells, regulates the muscle proliferation and differentiation through the transcriptional co-activators Yes-associated protein (Yap) and transcriptional co-activator with PDZ-binding motif (Taz). We found approximately 118 DMGs in the Hippo signaling pathway, indicating its important role in regulating muscle proliferation and differentiation in CGC. Wnt signaling pathway is known to be important for muscle growth and development as it regulates the differentiation of muscle stem cells (satellite cells) through regulating the expression of myogenic regulatory factors (MRFs), which are essential for myogenic lineage progression (Maltzahn et al., 2012). Wnt/β-catenin signaling has been reported to lead to unscheduled muscle progenitor proliferation in zebrafish (Tee et al., 2009). The Wnt signaling pathway with approximately 121 genes was differentially methylated between CGC and OGC muscles, indicating their potential roles in the regulation of muscle structure and development. In addition, mTOR signaling pathway has been reported to regulate myofiber differentiation and muscle cell hypertrophy (Glynn et al., 2008). JAK-STAT signaling pathway is also recognized as an essential pathway needed for muscle development and plays a crucial role in proliferation and differentiation of myoblasts through regulating JAK/STAT signaling cascade (Jang & Baik, 2013). Taken together, it was suggested that all of the above listed pathways might be tightly associated with muscle structure and development in crisp grass carp with extensive epigenetic modification. In addition, two immune-related pathways in promoter regions were observed, such as Inflammatory mediator regulation of TRP channels (93 genes) and Fc gamma R−mediated phagocytosis (86 genes), which is likely to be associated with the declined immunity and increased mortality of crisp grass carp fed with faba bean (Gan et al., 2017; Yu et al., 2017).

To further reveal epigenetic mechanisms underlying the muscle textural improvement in crisp grass carp, we performed an association analysis of DMGs and DEGs and generated a protein–protein interaction network of 34 overlap genes. This network included four parts: muscle fiber hyperplasia, collagen synthesis, oxidative response and immune response.

In fish, hyperplasia (increased myofiber number) and hypertrophy (increased myofiber size) are two important ways of muscle growth (Rowlerson et al., 1997). Previous studies have reported that the muscle growth and hardness improvement of crisp grass carp are the result of muscle fiber hyperplasia, but not hypertrophy (Johnston et al., 2000; Yu et al., 2017). In the present study, 13 key regulatory genes related to muscle fiber hyperplasia were found in following pathways: MAPK signal pathway (*myod* and *myog*), PI3K-Akt signaling pathway (*hsp90*), Hippo signaling pathway (*tgf-β1*, *smad3* and *smad4*), Wnt signaling pathway (*gsk3b*, *wnt8a*, *wnt11* and *axin2*), Jak-STAT signaling pathway (*stat1*, *stat2* and *jak2*). These results indicated that these pathways might play an important regulatory role in muscle development through DNA methylation.

MyoD, a member of the myogenic regulatory factor family, regulate myogenic progenitor cell activation and proliferation (Reuveni1 et al., 1999). Also, MyoG has been reported to be involved in muscle cell proliferation and the formation of multinucleated muscle fiber *via* promoting the formation of new muscle fiber (Hasty et al., 1993). HSP90 is a key member of molecular chaperone family, and it plays a crucial role in muscle cell proliferation for promoting myosin folding and assembling into myofibril (Du et al., 2008). TGF-β has been reported to promote vascular smooth muscle cell proliferation through the Smad3 and extracellular signal-regulated kinase mitogen-activated protein kinases pathways (Suwanabol et al., 2012). Wnt singaling pathway related genes (*gsk3b*, *wnt8a*, *wnt11* and *axin2*) is essential for promoting satellite cell proliferation and generate new myofibers (Girardi & Le-Grand, 2018). *Stat1*, *stat2* and *jak2* signaling are known to be involved in myoblast proliferation and differentiation during myogenesis (Trenerry, Gatta & Smith, 2011). Therefore, it can be inferred that these genes might play a critical role in muscle hardness improvement by promoting muscle fiber hyperplasia. Importantly, as previously reported, the hypermethylation within promoter regions tends to inhibit gene expression (Jones, 2012). Gene methylation analysis showed that, apart from *myod* and *smad4*, the methylation levels of these genes in crisp grass carp were lower than those in ordinary grass carp, which was negatively related to their expression level, suggesting that other regulatory mechanisms of muscle-specific transcriptional regulation are involved, since some studies have reported that DNA methylation is not the only factor affecting gene expression, and environmental factors, histones and transcription factors can also influence epigenetic status (Down et al., 2008). Taken together, our results suggest that DNA methylation may be involved in regulating muscle fiber hyperplasia in crisp grass carp by influencing the related genes expression, contributing to the muscle-hardening of crisp grass carp. Furthermore, previous study also showed that muscle hardness increase of the crisp grass carp were positively correlated with the deposition of collagen (Yu et al., 2014). In fish, the transforming growth factor β (TGF-β/Smads) pathway is a classic signaling for regulating collagen synthesis and deposition (Rong et al., 2022). TGF-β1, an important member of TGF-β family, has been reported to stimulate collagen synthesis in cell proliferation (Xu et al., 2018). Smad4 has been shown to up-regulate the synthesis type I collagen in crisp grass carp *via* TGF-β1/Smads pathway (Yu et al., 2019). In this study, the genes of *tgf-β1* and *smad4* were hypomethylated within the promoter regions in crisp grass carp, and the expression of these genes showed an opposite trend to methylation level. Thus, it was suggested the regulatory role of DNA methylation of *tgf-β1* and *smad4* in the collagen synthesis in the muscle of crisp grass carp, which is closely related to the muscle hardness improvement. Notably, the genes of *col1a1, col1a2, col1a3, col4a1 and col18a1* also showed higher expression level in crisp grass carp with hypomethylation in promoter regions, further confirming the key role of DNA methylation in regulating type I collagen expression and muscle hardness improvement in crisp grass carp.

Previous study found that faba bean significantly increased reactive oxygen species (ROS) levels in skeletal muscle of crisp grass carp, which has been proved to be responsible for the muscle hardness improvement in crisp grass carp (Yu et al., 2020; Chen et al., 2021). In this study, six genes related to oxidative response were found to be differentially methylated in crisp grass carp, including *g6pd, gst, gss, cat, prdx6* and *pex7*. G6PD has been reported to catalyse the rate-limiting step in the pentose phosphate pathway and produce NADPH, which not only protect cells against oxidative stress through preserving the reduced GSH but also contributes to maintaining the active form of CAT (Cappellini & Fiorelli, 2008). Moreover, PRDX6, a key member of the peroxiredoxin (PRDX) family, provides cytoprotection against ROS-induced oxidative stress (Fatma et al., 2008). Thus, the decreased expression of genes (*g6pd, gst, gss, cat, prdx6* and *pex7*) may promote the generation of ROS, resulting in improving muscle texture. Nevertheless, the differentially methylated levels in promoter regions of these oxidative response-related genes are not consistent (the genes of *gst and pex7* exhibited hypomethylation, whereas the genes of *g6pd*, *gss*, *cat* and *prdx6* exhibited hypermethylation), suggesting that DNA methylation is involved in transcriptional regulation in a more complex and dynamic manner. Generally, these results indicate that DNA methylation might participate in regulation of muscle texture by influencing the expression of oxidative response-related genes.

As previously reported, prolonged faba bean feeding history could lead to decreased immunity and increased mortality of grass carp (Gan et al., 2017; Yu et al., 2017). Consistent with this phenomenon, we found that many immune-related genes were differentially methylated in crisp grass carp, including *nfkb1*, *tnf-α*, *MHC-I*, *MHC-II*, *ifn-γ*, *il-4*, *il-10*, *il-12*, *il-21*, *il-23*. Among them, NF-κB (transcription factor nuclear factor kappa B), a major transcription regulator of immune response, regulate numerous genes involved in immune and inflammatory responses (Tsoulfas & Geller, 2001). NF-κB activity is stimulated by proinflammatory cytokines (*e.g*., TNF-α) when upon recognition of pathogen infections or tissue damage (Bonizzi & Karin, 2004). In this study, both *nfkb1* and *tnf-α* were hypomethylated in the promoter region of crisp grass carp, and the expression of *nfkb1* and *tnf-α* showed opposite trendency to methylation level, suggesting the DNA methylation of *NF-κB* and *tnf-α* in promoter region might play an important role in activating immune and inflammatory responses by regulating the expression level of *nfkb1* and *tnf-α*. Furthermore, MHC-I and MHC-II are two cell surface proteins essential for the acquired immune system for antigen presentation to recognize foreign molecules in vertebrates and can promote the development and expansion of T cells (Huang et al., 2005). In present study, the immune-related genes (*MHC-I* and *MHC-II*) exhibited hypermethylation in promoter region and decreased expression levels in crisp grass carp, which might indicate the immune disorder and decreased immunity of crisp grass carp. Therefore, these results suggested that different immune response levels between CGC and OGC may be mediated by DNA methylation. Notably, the activation of inflammation and oxidative stress have been reported to be involved in muscle atrophy, demonstrated by decreased muscle fiber diameter (Costamagna et al., 2015; Englund et al., 2001; Song et al., 2022). Moreover, the activation of NF-κB could lead to proinflammatory cytokine production such as TNF-α, IL-12 and IFN-γ, which plays an essential role in muscle atrophy (Thoma & Lightfoot, 2018). In this study, the expression of these proinflammatory cytokine genes were up-regulated with hypomethylation in the promoter region. Also, the hypomethylation of several anti-inflammatory cytokins genes (*il-4*, *il-10*, *il-21*, *il-23*) in promoter region might be responsible for their down-regulated expression levels. It has been reported that those anti-inflammatory cytokines genes (*il-4*, *il-10*, *il-21*, *il-23*) plays a critical role in regulating inflammation response *via* controlling the proinflammatory cytokine response (Schett, 2011). Therefore, it is suggested that DNA methylation might be involved in the occurrence of muscle inflammation which leads to a decreased in muscle fiber diameter. Similarly, some studies also found that dietary faba bean could reduce the diameter of muscle fibers in grass carp, tilapia and Yellow River carp (Yu et al., 2014; Lun et al., 2007; Song et al., 2022).

## Conclusion

In the present study, we performed a comprehensive analysis of DNA methylation profiles between crisp and ordinary grass carp, and many DMGs containing DMRs in promoter regions were identified. Our results show that crisp grass carp exhibit different DNA methylation patterns from ordinary grass carp after feeding faba bean, which likely affect the expression of genes related to muscle development. Specifically, we identified, in total, 34 key genes related to muscle development, 12 of which (*myod*, *myog*, *hsp90, smad3, smad4*, *gsk3b*, *wnt8a*, *wnt11*, *axin2*, stat*1*, *stat2*, *jak2*) were involved in muscle fiber hyperplasia, 6 of which (*tgf-β1*, *col1a1*, *col1a2*, *col1a3*, *col4a1*, *col18a1*) were associated with collagen synthesis in crisp grass carp, 6 of which (*g6pdh*, *gst*, *gss*, *cat*, *prdx6*, *pex7*) were related with oxidative response, and 10 of which (*nfkb1*, *tnf-α*, *ifn-γ*, *il-4*, *il-10*, *il-12*, *il-21*, *il-23*, *MHC-I*, *MHC-II*) were linked to immune response. The difference of methylation levels in the key genes might lead to the expression difference, further resulting in the increase of muscle hardness in crisp grass carp. Overall, this study of DNA methylation provided new clues for the epigenetic mechanisms regulating fish muscle quality, which might contribute to developing new strategies for the muscle quality improvement in other aquaculture fish species by nutritional programming.

## Supplemental Information

10.7717/peerj.14403/supp-1 Supplemental Information 1 The methods of DNA extraction and whole-Genome Bisulfite Sequencing.

10.7717/peerj.14403/supp-2 Supplemental Information 2 Raw data for textural quality of muscle.

10.7717/peerj.14403/supp-3 Supplemental Information 3 Differentially methylated genes (DMGs) in promoter regions.

10.7717/peerj.14403/supp-4 Supplemental Information 4 Differentially expressed genes (DEGs) of grass carp.

10.7717/peerj.14403/supp-5 Supplemental Information 5 Detailed methods and parameters of data analysis.

10.7717/peerj.14403/supp-6 Supplemental Information 6 Author Checklist - Full.

## Additional Information and Declarations

### Data Availability

The following information was supplied regarding data availability: The sequenced raw data is available at the NCBI Short Reads Archive (SRA): PRJNA835315.
