# Supplementary material for: Comparative genome-wide methylation analysis reveals epigenetic regulation of muscle development in grass carp (Ctenopharyngodon idellus) fed with whole faba bean
Source: PeerJ. 2022 Nov 21;10:e14403. doi: 10.7717/peerj.14403 (PMC9686415; doi:10.7717/peerj.14403)
Supplement: Supplemental Information 1 [file peerj-10-14403-s001.pdf]

# DNA Extraction SOP For Animal Tissue

| | |
|---|---|
| Document NO.: | SOP-SMM-J001 |
| Version NO.: | A0 |
| Effective Date： | 2016-4-15 |

## 1 Introduction

A phenol-chloroform extraction is a liquid-liquid extraction. A liquid-liquid extraction is a method that separates mixtures of molecules based on the differential solubilities of the individual molecules in two different immiscible liquids. Liquid-liquid extractions are widely used to isolate DNA.

## 2 Experimental Procedures

A. Pour 1ml lysis buffer.

B. For tissue samples, grind about 200mg with liquid nitrogen into powder and transfer the powder samples into the 2 ml tube contain of 1ml lysis buffer.

C. Incubate the sample at 56℃ in a water incubator for 30~180min. Mix by inversion every 5-10min time period.

D. Centrifuge at 16700×g for 10 minutes after cooling to room temperature.

E. Transfer the supernatant to a new 2.0ml tube, add equal volume of supernatant of Chloroform / isoamyl alcohol(24:1). Gently invert each 3-5min to mixed liquid no obvious boundaries.

F. Transfer the aqueous phase to a new 1.5mL tube; add equal volume of supernatant of isopropyl alcohol. Gently invert each 3-5min to mixed liquid no obvious boundaries.

G. Centrifuge at 16700×g for 10 minutes.

H. Transfer the aqueous phase to a new 1.5mL tube; add 2/3th volume of supernatant of isopropyl alcohol (add $1/10^{th}$ volume of 3M sodium acetate if necessary). Gently mix by inverting at least 3 times and place at -20℃ for 2 hours for precipitation.

I. Centrifuge at 16700×g for 10 minutes and remove the supernatant

J. Wash the RNA pellet with 1 ml 75% cooling ethanol. Re-suspend the pellet and centrifuge at 16700×g for 5 minutes at 4℃ and remove the supernatant

K. Wash the RNA pellet with 500ul 75% cooling ethanol. Re-suspend the pellet and centrifuge at 16700×g for 5 minutes at 4℃ and remove the supernatant.

L. 16700×g for 30~60s, Completely remove the ethanol without disturbing the pellet

M. Air-dry the RNA pellet in the biosafety cabinet

N. Add 25μL~100μL of TE Buffer to dissolve the DNA pellet. If a higher concentration of DNA is desired, 25μL of TE Buffer should be used.

## Appendix 1 the list of Reagents and Materials

### 1-1 Reagents

| Reagent Name | **Model** | **Manufacture** |
|---|---|---|
| Proteinase K | GPK003001-100mg | GPROAN |
| Absolute Ethanol | AR 500mL | Sinopharm |
| RNase A | 10mg | FERMENTAS |
| Guanidinium chloride | GB0242-500g | BBI |
| SDS | SS0228-1kg | BBI |

## Appendix 2 the list of Equipment

| Equipment Name | **Model** | **Manufacture** |
|---|---|---|
| Thermomixer comfort | Thermomixer comfort | Eppendorf |
| Digital thermostat water bath | HH-8 | Jingda |
| Centrifuge | 5427R | Eppendorf |
| Biological safety cabinets | Airstream | ESCO |

---------------------------------------------------------------END---------------------------------------------------------------
