# Supplementary material for: Comparative genome-wide methylation analysis reveals epigenetic regulation of muscle development in grass carp (Ctenopharyngodon idellus) fed with whole faba bean
Source: PeerJ. 2022 Nov 21;10:e14403. doi: 10.7717/peerj.14403 (PMC9686415; doi:10.7717/peerj.14403)
Supplement: Supplemental Information 5 [file peerj-10-14403-s005.pdf]

Methods

1 Experiment Pipeline

For normal ***WGBS*** library constructing, the DNA was fragmented by sonication using a Bioruptor (Diagenode, Belgium) to a mean size of approximately 250 bp, followed by the blunt-ending, dA addition to 3'-end, finally, adaptor ligation (in this case of methylated adaptors to protect from bisulfite conversion), essentially according to the manufacturer's instructions. Ligated DNA was bisulfite converted using the EZ DNA Methylation-Gold kit (ZYMO). Different ***Insert size*** fragments were excised from the same lane of a 2% TAE agarose gel. Products were purified by using QIAquick Gel Extraction kit (Qiagen) and amplified by PCR. At last, Sequencing was performed using the HighSeq4000 or other Illumina platforms.

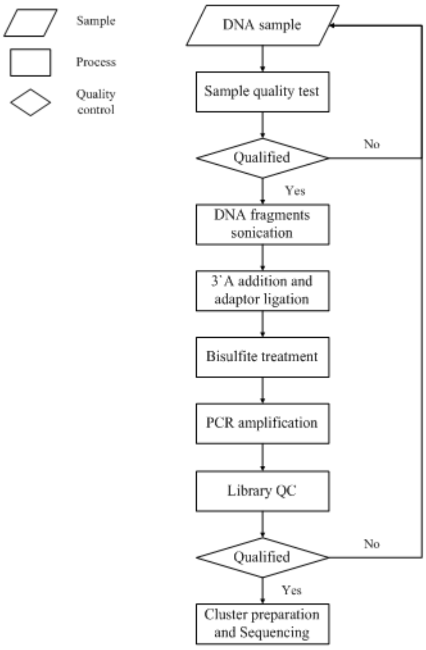

Figure 1 WGBS experimental process. <

After treatment with sodium bisulfite, unmethylated cytosine residues are converted to uracil whereas 5-methylcytosine (5mC) remains unaffected. After PCR amplification, uracil residues are converted to thymine. DNA methylation status can be determined by direct PCR sequencing or cloning sequencing.

2 Bioinformatics Pipeline

After getting ***Raw data*** , bioinformatics analysis will be performed according to the client appoints in the contract. Figure2 demonstrates a complete pipeline for ***WGBS***

深圳华大基因股份有限公司 400-706-6615 © BGI All Rights Reserved.

projects.

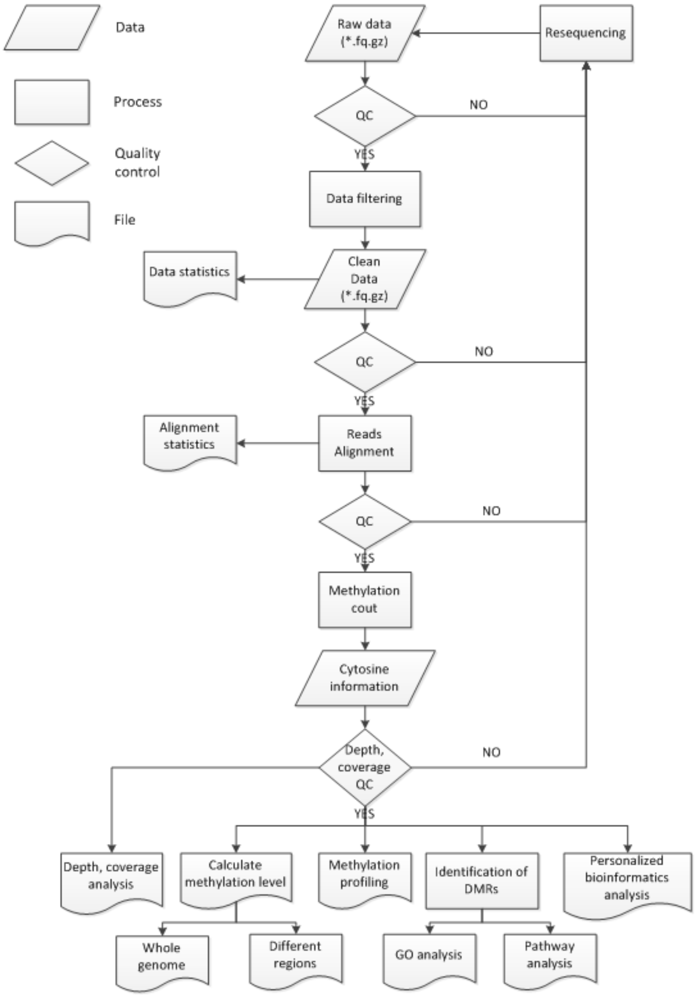

Figure 2 Bioinformatics analysis pipeline. <

After sequencing data was delivered, we did data filtering first, which could remove those low-quality data, and get clean data. Then, we mapped clean data to the reference genome when we made sure the data quantity of clean data was sufficient. Also, we needed to make a quality test about the alignment. And then we used those uniquely mapped data to get methylation information of the cytosine through the whole genome after we made sure that the alignment result was qualified. Later, we used the cytosine information for standard bioinformatics analysis and personalized bioinformatics analysis.

3 Data Filtering

Data filtering includes removing adaptor sequences, contamination and low-quality reads from raw reads. These reads were analyzed by BGI programs. Low-quality reads include two types, and the reads meet anyone of the two conditions will be removed:

1) Unknown bases are more than 10%;

2) The ratio of bases whose quality was less than 20 was over 10%.

After filtering, the remaining reads are called "clean reads" and stored as FASTQ format [2](see FASTQ format in help page).

深圳华大基因股份有限公司 400-706-6615 © BGI All Rights Reserved.

4 Reads Alignment

After filtering, the ***Clean data*** was then mapped to the reference genome by ***BSMAP***, and then remove the duplication reads and merge the mapping results according to each library. Here we calculate the ***Mapping rate*** and bisulfite ***Conversion rate*** of each sample.

Reads Alignment process

BSMAP parameters for PE reads: ***BSMAP*** -a filename_1.clean.fq.gz -b filename_2.clean.fq.gz -o filename.sam -d ref.fa

-u -v 8 -z 33 -p 4 -n 0 -w 20 -s 16 -f 10 -L 100

If use Hiseq2000, -z should be set 64;

samtools parameters:

samtools view -S -b -o filename.bam filename.sam

samtools sort -m 2000000000 filename.bam filename.sort

samtools index filename.sort.bam

5 Methylation level

Methylation level was determined by dividing the number of reads covering each mC by the total reads covering that cytosine[6], which was also equal the mC/C ratio at each reference cytosine[1]. The formula is showed as following figure:

$$Rm_{average} = \frac{\mathrm{Nm_{all}}}{\mathrm{Nm_{all}} + \mathrm{Nnm_{all}}} * 100\%$$

Nm represents the reads number of mC, while Nnm represents the reads number of non-methylation reads.

6 DMR detection

Putative ***DMRs*** were identified by comparison of the sample1 and sample2 methylomes using windows that contained at least 5 CpG(CHG or CHH) sites with a 2-fold change in ***Methylation level*** and Fisher test p value <= 0.05. In addition, we require that both tissues should not be hypomethylated in DMR discovery. Two nearby ***DMRs*** would be considered interdependent and joined into one continuous DMR if the genomic region from the start of an upstream DMR to the end of a downstream DMR also had 2-fold ***Methylation level*** differences between sample1 and sample2 with a p value <= 0.05. Otherwise, the two ***DMRs*** were viewed as independent. After iteratively merging interdependent ***DMRs***, the final dataset of ***DMRs*** was made up of those that were independent from each other.

7 Degree of difference in methylation level

We need to calculate the degree of difference of a ***methyl-cytosine*** (***mCG***, ***mCHG***, ***mCHH***) between two samples while comparing ***Methylation level*** of DMR in different samples by CIRCOS. The formula is showed as following figure:

$$\text{degree of difference} = \frac{\log_2 \mathrm{Rm1}}{\log_2 \mathrm{Rm2}}$$

深圳华大基因股份有限公司 400-706-6615 © BGI All Rights Reserved.

Rm1、Rm2 represent the ***Methylation level*** of ***methyl-cytosine*** for sample1 and sample2 respectively. 0.001 will replace Rm1 (or Rm2) while it is 0[9].

8 Gene Ontology Annotation

Gene Ontology (***GO***), which is an international standard gene functional classification system, offers a dynamic-updated controlled vocabulary, as well as a strictly defined concept to comprehensively describe properties of genes and their products in any organism. ***GO*** has three ontologies: molecular function, cellular component and biological process. The basic unit of ***GO*** is ***GO*** -term. Every ***GO*** -term belongs to a type of ontology.

GO enrichment analysis provides all ***GO*** terms that significantly enriched in a list of DMR-related genes, comparing to a genome background, and filter the DMR-related genes that correspond to specific biological functions. This method firstly maps all DMR-related genes to ***GO*** terms in the database (http://www.geneontology.org/), calculating gene numbers for every term, then uses hypergeometric test to find significantly enriched ***GO*** terms in the input list of DMR-related genes, based on ' ***GO*** ::TermFinder' (http://www.yeastgenome.org/help/analyze/go-term-finder), we have developed a strict algorithm to do the analysis, and the method used is described as follows:

$$P = 1 - \sum_{i=0}^{m-1} \frac{\binom{M}{i}\binom{N-M}{n-i}}{\binom{N}{n}}$$

Where N is the number of all genes with ***GO*** annotation; n is the number of DMR-related genes in N; M is the number of all genes that are annotated to certain ***GO*** terms; m is the number of DMR-related genes in M. The calculated p-value goes through Bonferroni Correction[10], taking corrected p-value ≤ 0.05 as a threshold. ***GO*** terms fulfilling this condition are defined as significantly enriched ***GO*** terms in DMR-related genes. This analysis is able to recognize the main biological functions that DMR-related genes exercise.

9 KEGG Pathway Enrichment

Pathway-based analysis helps to further understand genes biological functions. ***KEGG*** [11](the major public pathway-related database) is used to perform pathway enrichment analysis of DMR-related genes. This analysis identifies significantly enriched metabolic pathways or signal transduction pathways in DMR-related genes comparing with the whole genome background. The calculating formula is the same as that in ***GO*** analysis. Here N is the number of all genes that with ***KEGG*** annotation, n is the number of DMR-related genes in N, M is the number of all genes annotated to specific pathways, and m is the number of DMR-related genes in M.

深圳华大基因股份有限公司 400-706-6615 © BGI All Rights Reserved.
